# The Use of National Cancer Registry Data for Breast Cancer Family History Assessment in Premenopausal Women

**DOI:** 10.3390/jcm13154473

**Published:** 2024-07-31

**Authors:** Gabriel Chodick, Barbara G. Silverman, Lital Keinan-Boker

**Affiliations:** 1 School of Public Health, Tel Aviv University, Tel Aviv-Yafo 6997801, Israel; barbara.silverman@moh.gov.il; 2 Israel National Cancer Registry, Israel Center for Disease Control, Ministry of Health, Tel-Hashomer, Ramat Gan 5262000, Israel; lital.keinan2@moh.gov.il; 3 School of Public Health, University of Haifa, Haifa 3498838, Israel

**Keywords:** breast cancer, BRCA, family history, Israel

## Abstract

**Background**: Population-based cancer registries are the best source of information to measure cancer burden. However, little is done to use this information for individual cancer risk assessment. In this study, we aimed at identifying women at high risk of breast and ovarian cancer using data on family history of cancer from the Israel national cancer registry. **Methods**: We used the family history assessment tool (FHAT) to score all females, 26 to 45 years of age, in a 2.6-million-member health provider in Israel (Maccabi Healthcare Services). Data on breast, ovarian, prostate, and pancreatic cancer history among the participants and their parents (identified using the national census) were retrieved from the national cancer registry. These data were used to calculate individual FHAT scores. **Results**: A total of 377,931 eligible women were included in the analysis. A relevant family history of cancer was detected in 20,386 (5.4%), with FHAT scores ranging from 1 to 16. FHAT score was higher in older women and among those with a history of breast cancer. Among women aged 35–39, an FHAT score of 10 or above was associated with an OR of 15.23 (95%CI: 7.41–28.19) for breast cancer compared to women with an FHAT of 0. **Conclusions**: Using individual-level data from national cancer registries may assist in detecting women with a relevant family history of cancer.

## 1. Introduction

Breast cancer (BC) ranks as the most common cancer and the leading cause of cancer related mortality among Israeli women [1]. Meanwhile, ovarian cancer (OC) stands as the most lethal gynecological malignancy [2]. Among Ashkenazi Jewish (AJ) females, who constitute approximately 25% of the Israeli population, 1 in 40 is a carrier of one of three founder variants in the BRCA (breast cancer) gene: BRCA1_185delAG, BRCA1_5382insC, and BRCA2_6174delT [3,4]. Carriers of BRCA variant face lifetime risks of BC and OC of 69–72%  for BC, and 17–44% for OC [5]. It has been estimated that these BRCA variants are estimated to account approximately 10% of BC and 41% of OC within this population [3].

BC and OC are largely preventable if pathologic BRCA variants are identified prior to cancer development. Preventive risk management strategies may include MRI/mammographic screening, risk-reducing mastectomy, risk-reducing salpingo-oophorectomy, and pre-implantation genetic diagnosis [6]. In 2020, the Israeli Ministry of Health initiated a publicly funded BRCA1/2 carrier screening program for AJ women, without requiring pretesting genetic counseling. Adult Israeli women with full or partial AJ origin (based on self-report) and with no personal history of breast, ovarian, or pancreatic cancer are encouraged to undergo screening. However, by mid-2022 only a small fraction (3%) of eligible women had undergone screening for BRCA1/2 pathogenic variants [7].

Family history of cancer remains an important element of clinical decision support tools used to help identify candidates for hereditary cancer genetic testing [8,9]. However, a previous study in a large state-mandated health provider in Israel indicated that only 17% of female patients had a coded documentation of family history of cancer in their electronic health records (EHRs). Furthermore, only in 10%, family history was documented as a screening tool before any cancer diagnosis [10]. These findings suggest that self-reported information on family history in electronic records is ineffective for detecting patients at high-risk for genetic-related BC. Moreover, self-reported family history of cancer is of a sub-optimal validity with a sensitivity of only 61% for BC and 67% for OC [11]. In light of these limitations of self-reported information, exploration of better methods for assessing familial history of cancer is necessary to better identify individuals at increased risk for BC and OC.

Israel has a centralized, high-quality, community-based healthcare system. Over 98% of the population is covered by full health insurance and a majority has remained with the same linked EHR system for at least a decade. Israel’s four health providers have been accumulating medical data since late 1990s, nearly all of which is digitized. In addition, Israeli maintains a national cancer registry to which reporting of most types of cancer has been mandatory since 1982. The purpose of this study was to assess whether the data of the Israel national cancer registry can be effectively utilized to assess relevant family history of cancer and to identify adult females at increased risk for genetically mediated BC and OC.

## 2. Materials and Methods

### 2.1. Study Population

The study was conducted using the data of Maccabi Healthcare Services, a state-mandated health provider in Israel. Carrying BRCA1/2 pathological variants is much more likely in women diagnosed with breast cancer ≤ 45 years of age compared to those diagnosed in older ages [12]. Therefore, included in this analysis were all MHS females aged 26–45 in 2021 (born between 1976 and 1995). The mother and father of each member were identified from the Israel National Population Register using the coded unique identification number of the study participants. This also enabled the identification of sister/brothers within the study population. To protect data confidentiality, cross-linkage of family trios (mother–father–daughter) and data analysis were performed using TIMNA, a national research platform established by the Israeli government for conducting big-data research and combining de-identified health data from multiple health organizations.

### 2.2. Cancer Registry Data

Data on personal, parental, and first-degree relatives’ cancer histories were obtained from the Israel cancer register (INCR) and cross-linked with the target population using a coded unique identifier. The INCR was founded in 1960 and it covers the entire Israeli population (approximately 9 million in 2021). All malignant neoplasms are reported, excluding BCC and SCC; as well as benign neoplasms of the brain and nervous system are reported to the INCR. Sources of information include pathology reports, hospital discharge summaries, death certificates, and patient listings from cancer centers. Completeness of ascertainment has been estimated at 97% for solid tumors [13]. The registry currently includes information on approximately 800,000 people; 30,000 new cases are entered per year.

### 2.3. Assessing BC/OC Risk Score

We used the family history assessment tool (FHAT) [14] to quantify family history of cancer that is relevant for BC and OC risk. The FHAT model was selected because of its simplicity. Each member of the subject’s family who had been diagnosed with breast, ovarian, colon, or prostate cancer is scored (Supplement Table S1). Points are assigned based on the relationship to the woman, age at diagnosis, and number of primary cancers. For example, a woman with a sister that was diagnosed with BC (3 points) at age 31 years (4 points) and her maternal aunt was diagnosed with OC (3 points) at age 54 years (4 points), the total score for this woman’s family would be 14. A single individual may receive points from multiple categories. The model C-statistic for Ashkenazi patients (0.71; 95%CI: 0.66–0.77) is comparable to other commonly used models for estimating mutation probabilities of BRCA1 and BRCA2 [15].

### 2.4. Statistical Analysis

The standard FHAT cut-off value is set at 10 points [14]. Since our data lacked information on second- or third-degree relatives as well as on bilateral or multifocal tumors that limit FHAT potential maximal score, we used also a lower cut-off value of 8. In addition, we calculated the proportion of women with any relevant family history (FHAT of 1 or above). The odds ratios and Fisher’s exact 95% confidence intervals for BC by FHAT cut-off values and age group (26–34, 35–39, 40–45) were calculated. The study protocol has been approved by MHS and the Israel Ministry of Health. All analyses were performed using standard statistical software, IBM SPSS (Version 25, Armonk, NY, USA) and RStudio (RStudio 23.09.01, PBC, Boston, MA, USA).

## 3. Results

A total of 433,553 female members of MHS were identified (Supplementary Table S2). We excluded 5528 (1.3%) whose sex could not be accurately confirmed by the national census and 234 (0.1%) that were registered as males. Of the remaining 427,791 females, 49,860 (11.7%) who had no parents registered in the national census were also dropped from the study population, leaving a total of 377,931 eligible participants. Of these, 355,821 (94.1%) had a registered father and 373,855 (98.9%) had a registered mother.

### 3.1. Personal History of Cancer in the Study Population

The mean age in 2021 of the study participants was 35.58y (SD = 5.53 years). Among a total of 377,931 female members, a total of 1440 (0.4%) had a personal history of cancer, including 1030 with BC and 220 with OC (Table 1). The mean age at cancer diagnosis was 32.19, SD = 7.27.

### 3.2. FHAT Score and Risk of BC

**Figure 1:**
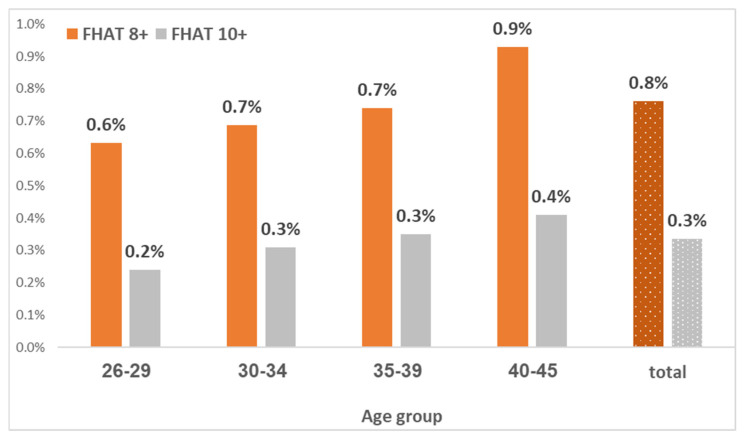
FHAT score by age group in Israeli women aged 26–45.

Among a total of 377,931 study females, 20,386 (5.4%) had any relevant family history of cancer (FHAT 1 or above), including 18,097 (4.8%) with a maternal history of BC, and 1524 (0.4%) with a maternal history of ovarian cancer. A total of 1271 (0.3%) females had an FHAT score of 10+, and 2876 (0.8%) had an FHAT score of 8+ (Figure 1). Therefore, the sensitivity of a positive FHAT score (10 or above) for breast cancer at the age groups of 26–34, 35–39, and 40–45 is 1.15% (1/87), 4.55% (11/242), and 1.59% (11/692), respectively (Table 2).

**Figure 2:**
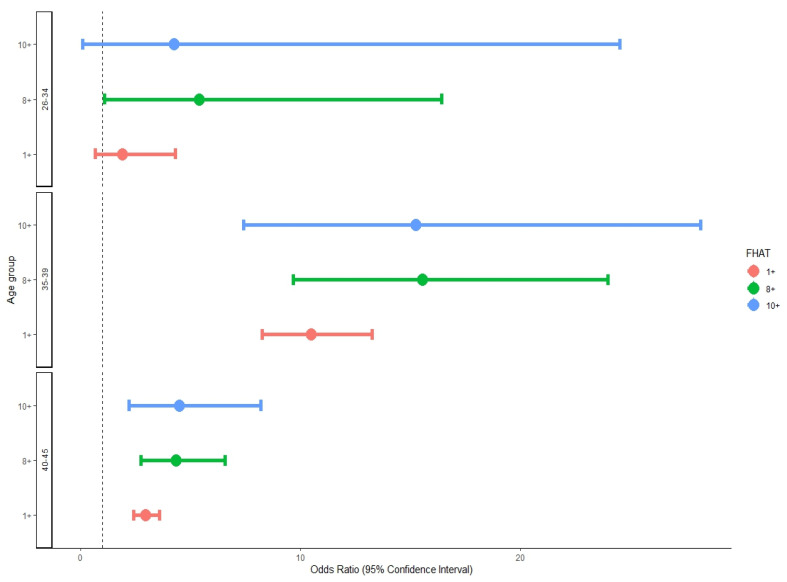
Odds ratio and 95%CI for breast cancer by age group and FHAT score.

Among women aged 35–39, an FHAT score of 10 or above was associated with an OR of 15.23 (95%CI: 7.41–28.19) for breast cancer compared to women with an FHAT of 0. A similarly high OR for breast cancer (OR = 15.55; 95%CI: 9.67–23.99) was calculated for women with an FHAT of 8 or above. In women aged 26–34 and 40–45, the OR for BC in women with an FHAT of 8 or above were 5.41 (1.09–16.42) and 4.34 (2.74–6.56), respectively (Figure 2).

## 4. Discussion

Accurate personal breast cancer risk assessment is essential for effective genetic screening and preventative interventions. Filtering women at a high risk of breast cancer in a time-sensitive manner is complicated because of being strongly age-dependent [16]. Given that self-reported family history is poorly documented in the Israeli medical system, we sought to explore using data from the Israel National Cancer Registry to detect women with a family history of cancer that would put them at increased risk of BC. Our results suggest that using the national cancer registry data, 7.2% of Israeli women aged 40–45 were found to have a family history of cancer that might increase their personal risk of breast cancer (an FHAT score of 1+). This aligns with a prevalence of 8.5% of women age 40–49 that reported on a positive family history in a previous national cross-sectional survey of the Israeli population [17]. To our knowledge, this approach of using national cancer registry data for risk assessment is novel in the literature.

The findings of the current analysis indicate that young adult women with a positive score on the FHAT, a standard genetic risk assessment tool, were substantially more likely to be diagnosed with BC compared to women with no relevant family history. The elevated likelihood was particularly high among women aged 35–39 where an FHAT score of 8 or above accounts for 0.7% of the general population and nearly 10% of women with BC. Therefore, the use of national cancer registry data may effectively improve screening efforts for genetic-related breast cancer in young adult women as well as for risk stratification in order to implement annual mammography and early detection of BC.

Currently, a BRCA1/2 carrier screening program is offered to AJ women in Israel irrespective of family history [7]. This may explain the relatively low carrier frequency of the three AJ founder variants in BRCA1/2 that were detected under this program (0.95%), compared to the reported frequency in previous studies (2.5%) [18]. This and the relatively poor uptake of the test calls into question the cost-effectiveness of the current screening policy. Nonetheless, the traditional genetic testing policy that has been restricted to individuals fulfilling family history eligibility criteria had a limited sensitivity, missing 60% of BRCA carriers (14). Additionally, this policy is characterized by a major underutilization due to limited awareness and access. In the US, it has been estimated that fewer than one in five individuals with a history of BC or OC the meet the National Cancer Comprehensive Network criteria, have undergone genetic testing [19]. Similarly, approximately 90% and 97% of BRCA carriers in the Jewish and the general population of the UK, remain unidentified, respectively [20]. The use of readily available historic data from national cancer registries may offer a new method for determining family history of young adult females and improve current gaps in BC/OC prevention and more efficient identification of pathogenic variants BRCA1/2 mutation female carriers.

There are some potential weaknesses in the present study. We did not have information about the history of BRCA1/2 tests as the cancer registry does not have access to the results of genetic testing. Therefore, we could not assess the validity of FHAT tools for genetics referral, but only against cancer occurrence. In addition, we lacked data on ethnic origin and thus were unable to distinguish between AJ, non-AJ, and Arab females. According to 2022 data from the largest state-mandated health provider in Israel (CHS), approximately 28% of females aged 26–45 are AJ. Therefore, the observed association between FHAT score and BC occurrence underestimates the relationship if analyses had been restricted to AJ. Of note, the CHS study results indicate that the carrier frequency of the three AJ founder variants in BRCA1/2 is 0.95% (95%CI: 0.77–1.18%) among all AJ groups, substantially lower than the reported frequency in a previous study in Israeli and in international studies (~2.5%) [21,22].

A population-based screening program for BRCA variants among AJ in Israel has been found to be more cost-effective compared with family history-based strategies [23]. The use of the cancer registry data to identify women with a significant family history may improve the implementation of these screening efforts and serve an example of using precision medicine for cancer screening. Adjustment of screening recommendations to personal risk level, rather than age-based population screening, could promote early diagnosis among young adult women while maintaining high specificity and improving its cost-effectiveness.

At a younger age fewer first-degree relatives are likely to have received a cancer diagnosis. Therefore, family history is age-dependent and thus may change over time. In our cohort, any relevant family history of cancer among the average-risk population increased from 4.1% at the age of 26–34 to 7.2% at age 40–45. It is therefore recommended that family history be updated periodically, especially among patients in their 30s and 40s.

## 5. Conclusions

In light of the underutilization of BRCA pathologic variant testing among eligible women in Israel, the poor documentation of family history in electronic records, and the sub-optimal validity of self-reported family history, we propose to seek avenues for utilizing the data of the INCR in order to increase awareness and uptake of BRCA testing among women at high-risk of BC and OC. This should be considered while adopting robust security measures, respecting patient autonomy, and ensuring compliance with regulatory data privacy standards.

## Figures and Tables

**Table 1:** Personal history of cancer among study participants by age group in 2021 (N = 377,931).

	26–34 y		35–39 y		40–45 y		Total	
	n	%	n	%	n	%	n	%
**Total**	182	100%	348	100%	910	100%	1440	100%
Breast	88	48.4%	243	69.8%	699	76.8%	1030	71.5%
Ovary	52	28.6%	55	15.8%	113	12.4%	220	15.3%
Colon	19	10.4%	19	5.5%	50	5.5%	88	6.1%
Stomach, intestines, rectum	13	7.1%	10	2.9%	22	2.4%	45	3.1%
Pancreas	2	1.1%	8	2.3%	13	1.4%	23	1.6%
Digestive organs, uns.	4	2.2%	4	1.1%	2	0.2%	10	0.7%
Lymphoma	1	0.5%	1	0.3%	3	0.3%	5	0.3%
Reticulosarcoma	0	0.0%	2	0.6%	2	0.2%	4	0.3%
Oral cavity	1	0.5%	0	0.0%	3	0.3%	4	0.3%
Carcinoid	1	0.5%	1	0.3%	2	0.2%	4	0.3%
Hepatic flexure	0	0.0%	2	0.6%	1	0.1%	3	0.2%
Skin of trunk, in situ	0	0.0%	2	0.6%	0	0.0%	2	0.1%
Female genital organs	1	0.5%	1	0.3%	0	0.0%	2	0.1%

**Table 2:** FHAT scores by breast cancer (BC) and ovarian cancer (OC) status in Israeli women aged 26–45.

	None		Breast Cancer		Ovarian Cancer		Either	
	n	%		%		%		%
**Age 40–45**								
FHAT 10+	434	0.40%	11	1.60%	1	0.90%	12	1.50%
FHAT 8+	984	0.90%	24	3.50%	2	1.90%	26	3.30%
FHAT 1+	7758	7.20%	129	18.60%	8	7.50%	137	17.10%
FHAT = 0	100,112	92.80%	563	81.40%	99	92.50%	662	82.90%
Total	107,898	100%	692	100%	107	100%	799	100%
**Age 35–39**								
FHAT 10+	328	0.30%	11	4.50%	1	1.80%	12	4.00%
FHAT 8+	701	0.70%	24	9.90%	2	3.60%	17	5.70%
FHAT 1+	5440	5.60%	41	16.90%	8	14.50%	47	15.80%
FHAT = 0	91,289	94.40%	201	83.10%	49	89.10%	250	84.20%
Total	96,729	100%	242	100%	55	100%	297	100%
**Age 26–34**								
FHAT 10+	485	0.3%	1	1.1%	0	0.0%	1	0.7%
FHAT 8+	1145	0.7%	3	3.4%	0	0.0%	3	2.2%
FHAT 1+	7002	4.1%	7	8.0%	1	2.0%	8	5.8%
FHAT = 0	165,103	95.9%	80	92.0%	49	98.0%	129	94.2%
Total	172,105	100%	87	100%	50	100%	137	100%

## Data Availability

Data are unavailable due to privacy restrictions.

## Supplementary Materials

The following supporting information can be downloaded at: https://www.mdpi.com/article/10.3390/jcm13154473/s1, Table S1: FHAT Scoring Method*. Table S2: Characteristics of female MHS members aged 26–45 in 2021 (N = 433,553).
