# Supplementary material for: The Use of National Cancer Registry Data for Breast Cancer Family History Assessment in Premenopausal Women
Source: J Clin Med. 2024 Jul 31;13(15):4473. doi: 10.3390/jcm13154473 (PMC11313154; doi:10.3390/jcm13154473)
Supplement: Supplementary file 1 [file jcm-13-04473-s001.zip › jcm-3098930-supplementary.pdf]

Supplementary Material

Supplement Table S1: FHAT scoring method*

**Risk Factor**		**Points**
**Breast &**	mother	10
**Ovarian cancer**	sibling	7
	$2^{nd}/3^{rd}$ degree	5
**Breast cancer**	mother	4
	sibling	3
	$2^{nd}/3^{rd}$ degree	2
	male add to above	2
**Age of onset**	20-29y	6
**(breast cancer)**	30-39y	4
	40-49y	2
	Pre (peri) menopausal	2
**Breast cancer**	Bilateral/multifocal tumor	3
**Ovary cancer**	mother	7
	sister	4
	$2^{nd}/3^{rd}$ degree	3
**Age of onset (ovary cancer)**	<40y	6
	40-60y	4
	>60y	2
**Age of onset (prostate cancer)**	<50y	1
**Age of onset (colon cancer)**	<50y	1

*Adopted from (14)

Supplement Table S2: Characteristics of female MHS members aged 26-45 in 2021 (N=433,553)

Vriable	Category	%
Residential region	North	18.6
	Center	65.6
	South	15.8
Socioeconomic status*	1 (lowest)	0.7
	2	3.4
	3	9.8
	4	12.3
	5	16.8
	6	19.6
	7	16.9
	8	10.8
	9	7.9
	10	2.4
Special populations**	Disability benefits	2.2
	Income support	1.8
	Students	10.3

*According to residential area data provided by Points business mapping Ltd., ramat gan, israe (https://points.co.il/en/points-location-intelligence/l) ** Israel National Insurance Institute report (https://www.btl.gov.il/Publications/survey/Documents/320/seker_320.pdf)
